# Histone Modifications Are Associated with Transcript Isoform Diversity in Normal and Cancer Cells

**DOI:** 10.1371/journal.pcbi.1003611

**Published:** 2014-06-05

**Authors:** Ondrej Podlaha, Subhajyoti De, Mithat Gonen, Franziska Michor

**Affiliations:** 1 Department of Biostatistics and Computational Biology, Dana-Farber Cancer Institute, and Department of Biostatistics, Harvard School of Public Health, Boston, Massachusetts, United States of America; 2 Department of Medicine, University of Colorado School of Medicine, Aurora, Colorado, United States of America; 3 Department of Biostatistics and Informatics, Colorado School of Public Health, Aurora, Colorado, United States of America; 4 Molecular Oncology Program, University of Colorado Cancer Center, Aurora, Colorado, United States of America; 5 Department of Epidemiology and Biostatistics, Memorial Sloan-Kettering Cancer Center, New York, New York, United States of America; Rutgers University, United States of America

## Abstract

Mechanisms that generate transcript diversity are of fundamental importance in eukaryotes. Although a large fraction of human protein-coding genes and lincRNAs produce more than one mRNA isoform each, the regulation of this phenomenon is still incompletely understood. Much progress has been made in deciphering the role of sequence-specific features as well as DNA-and RNA-binding proteins in alternative splicing. Recently, however, several experimental studies of individual genes have revealed a direct involvement of epigenetic factors in alternative splicing and transcription initiation. While histone modifications are generally correlated with overall gene expression levels, it remains unclear how histone modification enrichment affects relative isoform abundance. Therefore, we sought to investigate the associations between histone modifications and transcript diversity levels measured by the rates of transcription start-site switching and alternative splicing on a genome-wide scale across protein-coding genes and lincRNAs. We found that the relationship between enrichment levels of epigenetic marks and transcription start-site switching is similar for protein-coding genes and lincRNAs. Furthermore, we found associations between splicing rates and enrichment levels of H2az, H3K4me1, H3K4me2, H3K4me3, H3K9ac, H3K9me3, H3K27ac, H3K27me3, H3K36me3, H3K79me2, and H4K20me, marks traditionally associated with enhancers, transcription initiation, transcriptional repression, and others. These patterns were observed in both normal and cancer cell lines. Additionally, we developed a novel computational method that identified 840 epigenetically regulated candidate genes and predicted transcription start-site switching and alternative exon splicing with up to 92% accuracy based on epigenetic patterning alone. Our results suggest that the epigenetic regulation of transcript isoform diversity may be a relatively common genome-wide phenomenon representing an avenue of deregulation in tumor development.

## Author Summary

Traditionally, the regulation of gene expression was thought to be largely based on DNA and RNA sequence motifs. However, this dogma has recently been challenged as other factors, such as epigenetic patterning of the genome, have become better understood. Sparse but convincing experimental evidence suggests that the epigenetic background, in the form of histone modifications, acts as an additional layer of regulation determining how transcripts are processed. Here we developed a computational approach to investigate the genome-wide prevalence and the level of association between the enrichment of epigenetic marks and transcript diversity generated via alternative transcription start sites and splicing. We found that the role of epigenetic patterning in alternative transcription start-site switching is likely to be the same for all genes whereas the role of epigenetic patterns in splicing is likely gene-specific. Furthermore, we show that epigenetic data alone can be used to predict the inclusion pattern of an exon. These findings have significant implications for a better understanding of the regulation of transcript diversity in humans as well as the modifications arising during tumor development.

## Introduction

Molecular processes such as alternative splicing and transcription start-site switching are primary drivers of transcript diversity. About 95% of the ∼23,000 human genes are estimated to produce more than one mRNA isoform [1]. Beyond the genes with protein-coding potential, recent discoveries suggest that the approximately 8,000 large intergenic noncoding RNAs (lincRNAs) found in the human genome generate on average 2.3 isoforms per lincRNA locus [2].

The analysis of transcript diversity regulation has traditionally focused on splicing factors and RNA sequence features such as splicing enhancers and silencers [3], [4]. In recent years, however, experimental studies have expanded to include other regulatory factors such as histone modifications, suggesting that epigenetic features may have the ability not only to determine when and in which tissues certain genes are expressed, but also to influence how these transcripts are processed. Genome-wide analyses indicate that nucleosomes and histone modifications are not randomly distributed, but often coincide with exon boundaries [5]–[7]. This observation, combined with recent evidence that most events of alternative splicing in human cells occur co-transcriptionally [8], [9], strongly suggest a regulatory potential of histone marks [2], [10].

While the connection of epigenetic regulation with overall gene expression has largely been elucidated [11]–[14], it is much less clear whether and how epigenetic marks determine relative isoform abundance. Qualitative and quantitative models have been built to predict expression on the level of genes using histone modification enrichment information alone [15]. Interestingly, a quantitative prediction model based on histone modification enrichment outperforms models based on transcription factor binding [15]. However, a systematic evaluation of the association of epigenetic marks with transcription start-site switching and splicing frequency is still lacking in the literature. Work by Ernst et al. [16], [17], who classified chromatin states to functionally annotate the genome, identified a combination of histone modifications, which were associated with transcription start site and spliced exons. However, since in this work, the histone mark ChIP-seq tag counts were processed into binary presence and absence calls and since isoform abundance was not estimated from the expression data, the critical question remains whether different levels of epigenetic enrichment are associated with the rates of transcription start-site switching and splicing.

In addition to elucidating the epigenetic regulation of transcript diversity, further open questions remain. These questions pertain for instance to the genome-wide prevalence of epigenetic regulation of transcript diversity generated via alternative splicing or transcription start-site switching. Furthermore, it is unclear to what extent the involvement of epigenetic marks in the regulation of transcript diversity is gene-specific, ie. whether individual genes respond to different histone marks or whether there is a “universal” set of marks for alternative splicing. Several studies aimed at deciphering the association between histone modifications and alternative splicing on a genome-wide scale [18]–[22] but relied solely on gene annotation for the assignment of alterative splicing events rather than on a comprehensive transcription analysis [22], or with no more than three cell lines lacked the breadth of conditions analyzed [18], [19], [21]. Finally, the association of epigenetic patterning with transcript diversity in cancer cells has not been analyzed methodically in a genome-wide manner; however, understanding the prevalence of this phenomenon is of particular importance in cancer where cells are known to undergo vast epigenetic aberrations [23]. Indeed, epigenetically divergent regions in cancer cell lines are enriched for cancer-associated genes (**Module S1 in Text S1**).

**Figure 1:**
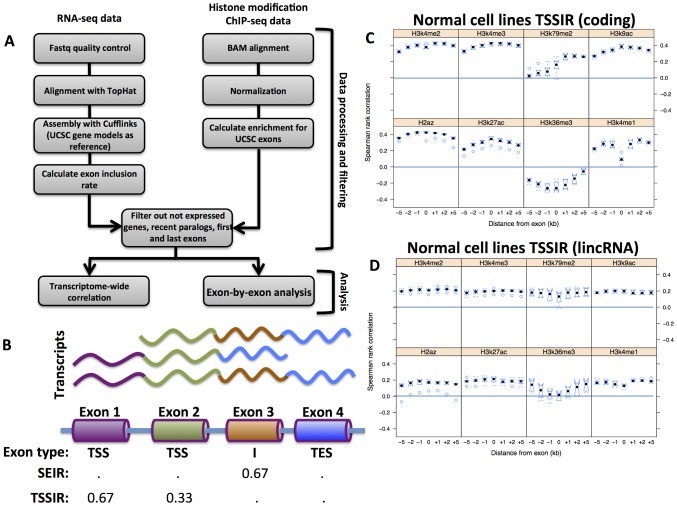
Analysis workflow and association between histone modification enrichment and transcription start site inclusion rate. (**A**) Schematic of the analysis workflow employed in this study. (**B**) The exon inclusion rate (SEIR and TSSIR) represents the proportion of transcripts of a given gene stemming from a given exon. In this example, three transcripts representing three different splice forms are generated from a single gene. The three isoforms are generated via two transcription start sites and one splicing event. Exon 1 is present in two transcripts, and since it is a transcription start site (TSSIR = 0.67). Exon 2 is present in three transcripts and is a transcription start site for one of the isoforms (TSSIR = 0.33). Exon 3 is present two transcripts and is spliced out in one isoform (SEIR = 0.33). Lastly, exon 4 is a transcription end site and is not considered in our analysis. (**C**) and (**D**) are correlations between transcription start site inclusion rate and enrichment of selected histone modifications in normal cell lines for protein coding genes and lincRNAs, respectively. Black dots represent median Spearman rank correlations between exon inclusion rate and H3K4me1, H3K4me2, H3K4me3, H3K9ac, H3K27ac, H3K79me2, H3K36me3, and H2az enrichments in normal cell lines. All correlation coefficients were transformed using a Fisher's transformation before plotting. Notches were calculated as 
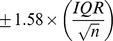
 where *IQR* stands for inter quartile range and *n* for sample size. Distances from exon represent genomic blocks of a given size from exon start (upstream regions) or exon end (downstream regions).

Here, we sought to perform a detailed study investigating the association between histone modification enrichments and the processes that influence isoform abundance – transcription start-site switching and splicing – on a genome-wide level (**Fig. 1A** **and** **Table 1**). We further developed a novel approach that identified a set of 840 genes for which transcription start-site switching and splicing was strongly associated with at least one epigenetic mark. We also showed that histone modification enrichment alone can predict exon splicing and transcription start-site switching with up to 92% accuracy in an independent sample set. Our work strongly suggests a broad-scale involvement of epigenetic factors in transcription start-site switching and alternative splicing.

**Table 1:** Summary of datasets used in this study.

Feature	Data type	Cell type	Source
Gene expression	RNA-seq	Gm12878, Hsmm, Huvec, Hepg2, Helas3, K562, H1hesc, Nhek, Nhlf	ENCODE
Histone modification	ChIP-seq for H3K4me1, H3K4me2, H3K4me3, H3K9ac, H3K9me1, H3K9me3, H4K20me1, H3K27ac, H3K27me3, H3K36me3, H3K79me2, H2az	Gm12878, Hsmm, Huvec, Hepg2, Helas3, K562, H1hesc, Nhek, Nhlf	ENCODE
lincRNA annotation	GTF	human genome version hg19	Ref. 2
Gene annotation	GTF	human genome version hg19	www.genome.ucsc.edu

ENCODE – data generated by the ENCODE consortium [8], available at http://genome.ucsc.edu/ENCODE/downloads.html.

GTF – gene transfer file format used for human genome annotation.

## Results

### Data sets and analyses

We examined RNA-seq data from nine human cell lines (Gm12878, Hsmm, Huvec, Hepg2, Helas3, K562, H1hesc, Nhek, Nhlf) (http://genome.ucsc.edu/ENCODE/), of which six were normal (Gm12878, Hsmm, Huvec, H1hesc, Nhek, Nhlf) and three were cancer cell lines (Hepg2, Helas3, K562). For all nine cell lines, we obtained information of the genome-wide patterns of the following twelve histone marks: H3K4me1, H3K4me2, H3K4me3, H3K9ac, H3K9me1, H3K9me3, H4K20me1, H3K27ac, H3K27me3, H3K36me3, H3K79me2, H2az.

Our analysis of the association between histone enrichment and transcript diversity utilized two different approaches: (i) a genome-wide approach, and (ii) an exon-specific approach. The genome-wide method analyzes each cell line individually and investigates all exons with a given characteristic (i.e. spliced, not spliced, transcription start site exon, etc.) at once, irrespective of the gene of origin. The exon-specific approach, in contrast, analyzes one exon at a time across multiple cell lines. The latter approach is able to identify candidate exons or genes with potential epigenetic regulation of transcription diversity and is analogous to an experimental setup in which each cell line represents an experimental condition (i.e. varying levels of histone modification enrichment) resulting in a particular exon inclusion or transcription start site outcome. The genome-wide approach requires a set of assumptions (see Discussion section); however, due to the large sample size of exons, it may uncover associations that would otherwise not be significant at a single gene level. With sufficiently many samples and sequencing depth, the patterns of associations uncovered by both approaches converge.

### The splicing exon inclusion rate and transcription start site inclusion rate

To assess the level of transcript diversity in the human genome, we analyzed RNA-seq data from nine human cell lines and quantified the abundance of specific mRNA isoforms for each protein-coding gene and lincRNA. We mapped and assembled the transcriptome of each cell line using the TopHat2 and Cufflinks2 softwares [24], [25], respectively, using merged UCSC reference annotation with lincRNA annotation from Cabili and colleagues [2] as a set of assembly models (see **Methods**). In order to minimize confounding issues, for instance with the misalignment of RNA-seq reads, we excluded paralogs that were more than 95% identical on the DNA sequence level. Exons were grouped into four categories: transcription start site, internal, transcription end site, or overlapping exons. Only internal and transcription start site exons were used for further analysis. The level of expression of an internal and transcription start site exon was quantified by calculating the splicing exon inclusion rate (SEIR, ranging from 0 to 1) and transcription start site inclusion rate (TSSIR, ranging from 0 to 1) respectively, both of which reflect the proportion of transcripts containing a given exon at a given gene locus (**Fig. 1B**). An SEIR of 0 implies that a given exon is always spliced in all expressed isoforms of a gene, whereas an SEIR of 1 implies that a given exon is always retained. A TSSIR of less than 1 signifies that a given exon occasionally represents the first exon of an expressed isoform, whereas a TSSIR of 1 indicates that a given exon serves as the transcription start site for all expressed isoforms. The SEIR and TSSIR measures therefore identify exons contributing to transcript diversity of a given gene.

### Transcriptome-wide association of histone mark enrichment with TSSIR and SEIR

We hypothesized that, if histone modification enrichment patterns play a significant role in transcript diversity, then the levels of transcription start-site switching and splicing should correlate with the enrichment levels of certain histone modifications within each cell line analyzed. We therefore investigated the transcriptome-wide association between histone mark enrichment and TSSIR and SEIR within each cell line. To address the possibility that transcript diversity in cancer cell lines is regulated differently as compared to that in normal cell lines, we quantified the level of association in the normal cell lines first and then assessed the degree of similarity in this pattern between normal and cancer cell lines. To this end, we determined the expression profiles as well as histone modification enrichment for all annotated exons of protein-coding genes and lincRNAs in the normal cell lines (**Methods**). Out of the twelve histone marks examined, seven (H3K4me1, H3K4me2, H3K4me3, H3K9ac, H3K27ac, H3K79me2, and H2az) showed a strong positive association with transcription start-site switching for both protein-coding genes and lincRNAs (**Fig. 1C and 1D**). Although the involvement of H3K4me2 and H3K4me3, H3K9ac, and H3K27ac in transcription initiation was expected given the findings of previous studies [16], [17], the presence of H3K79me2 and H2az was not anticipated. These results suggest that the transcription initiation of both protein-coding genes and lincRNAs is probably regulated via similar molecular mechanisms.

**Figure 2:**
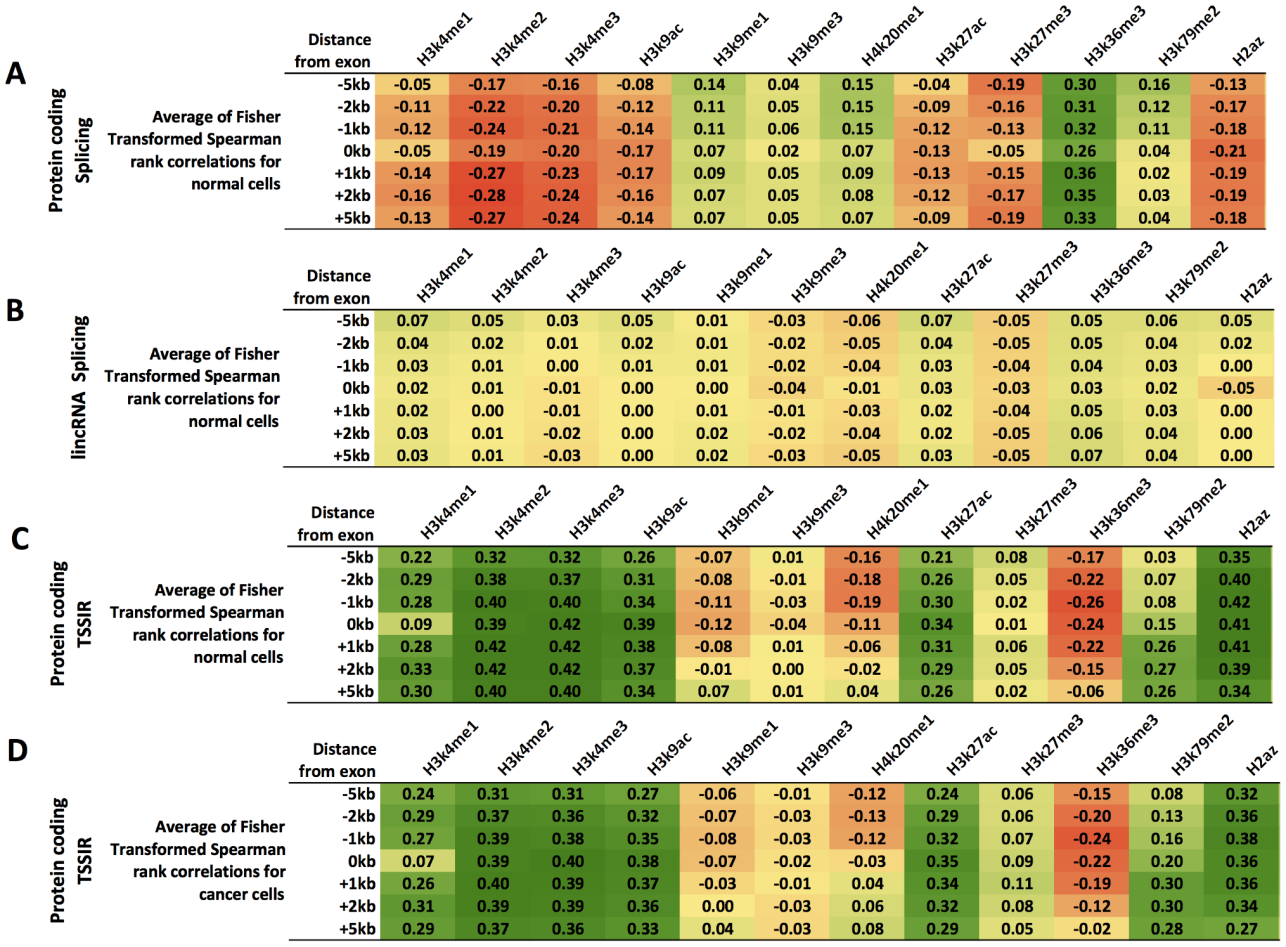
Comparison of epigenetic association between normal and cancer cell lines. We analyzed (**A–C**) six normal human cell lines (Gm12878, Hsmm, Huvec, H1hesc, Nhek, Nhlf) and (**D**) three cancer cell lines (Hepg2, Helas3, K562) for associations between transcription start site inclusion rate and splicing exon inclusion rate and histone modification enrichment for protein-coding genes (**A,C,** and **D**) and lincRNAs (**B**). Values represent the average of Fisher transformed Spearman rank correlations to enable direct comparison. Coefficients are color-coded, with red representing increasingly negative and green representing increasingly positive correlation. Distance from exon categories signifies a region relative to a given exon where histone enrichment was measured; 0 kb represents region within given exon boundaries, and 1 kb, 2 kb, and 5 kb signify regions from the exon boundary either upstream (negative) or downstream (positive).

The transcription profiles of the nine cell lines revealed that many protein-coding genes as well as lincRNAs undergo alternative splicing. Given the fact that transcription start-site switching occurs in a similar epigenetic background for protein coding genes as well as lincRNAs, we then sought to investigate whether splicing in protein-coding genes and lincRNAs is also associated with a similar set of histone marks. We found that splicing in protein-coding genes was most strongly positively correlated with the enrichment of H3K36me3 and negatively correlated with H3K4me2 and H3K4me3 (**Fig. 2A**). H3K36me3 has been previously found to mark actively transcribed regions and to regulate the splicing of FGFR2 [26], thus confirming our results. However, splicing of lincRNAs did not reveal any association with histone mark enrichment (**Fig. 2B**), suggesting that splicing of non-coding RNAs is either independent of the epigenetic background, involves sequence-specific regulation, and/or occurs post-transcriptionally.

We then aimed to investigate whether this pattern was consistent when taking into account exon number per gene, gene expression patterns, and genomic features such as simple repeats, microsatellites, and conserved elements. Controlling for these factors, the correlations between TSSIR and H3K4me2 as well as H3K9ac were very robust, varying for instance in the Gm12878 cell line between 0.35<ρ<0.37 for H3K4me2 (uncontrolled correlation ρ = 0.37) and between 0.35<ρ<0.38 for H3K9ac (uncontrolled correlation ρ = 0.37). Similarly, controlling for H3K9ac enrichment reduced the correlation between TSSIR and H3K4me2 by only 0.5%, and controlling for H3K4me2 enrichment reduced the correlation between TSSIR and H3K9ac by only 3.18%. These observations suggest that, while the interplay between transcript diversity and epigenetics probably involves many other factors, which might occlude the signal, the association between the SEIR and specific histone marks is genuine.

### Spatial patterns of correlation between histone enrichment, TSSIR, and SEIR

Recently, a study examining the alternative splicing of *CD45* showed that molecular interactions as far as 1 kb downstream of exon 5 affected its inclusion rate [27]. To investigate how epigenetic marks at a distance from exons influences transcript diversity on a genome-wide scale, we analyzed histone enrichment profiles at distances of 1 kb, 2 kb, and 5 kb immediately upstream and downstream of the exon locus (**Methods**). We identified pronounced differences in spatial patterns of correlation strength between the previously identified histone marks H3K4me1 and H3K79me2 and the TSSIR of protein-coding genes in normal cells (**Fig. 1C**). For example, the correlation between TSSIR and H3K4me1 at the exon locus was very weak (*z*_0 kb_ = 0.09) but rose to much higher levels as close as 1 kb upstream and downstream of the spliced exon (z_−1 kb_ = 0.28, z_1 kb_ = 0.28); this level of correlation was also observed for distances of 2 kb and 5 kb upstream and downstream of the exon (z_−5 kb_ = 0.22, z_−2 kb_ = 0.29, z_2 kb_ = 0.33, z_5 kb_ = 0.30). Interestingly, a very different spatial pattern was observed for the histone mark H3K79me2, for which the correlation between TSSIR and histone enrichment upstream and at the exon locus was weak (z_−5 kb_ = 0.03, z_−2 kb_ = 0.07, z_−1 kb_ = 0.08, z_0 kb_ = 0.15), but became much stronger at distances of 1–5 kb downstream of the exon (*z*_1 kb_ = 0.26, *z*_2 kb_ = 0.27, *z*_5 kb_ = 0.26). The spatial pattern of correlation between H3K4me1 enrichment and TSSIR for lincRNAs was less pronounced (**Fig. 1D**), showing lower levels of correlation at the exon locus compared to up- and downstream regions (z_−5 kb_ = 0.17, z_−2 kb_ = 0.18, z_−1 kb_ = 0.15, z_0 kb_ = 0.13, *z*_1 kb_ = 0.19, *z*_2 kb_ = 0.20, *z*_5 kb_ = 0.19).

The only spatial pattern evident for an association between histone enrichment and SEIR was observed for H3K36me3 (**Fig. 2A**). While the correlation outside the exon boundaries ranged from 0.30<z<0.36, the correlation at the exon locus itself was slightly diminished to z_0 kb_ = 0.26. It remains unclear which factors drive the spatial distribution of H3K36me3; for example, Luco et al. showed that H3K36me3 interacts with the FGFR2 pre-mRNA via the MRG15/PTB chromatin-adaptor complex, which regulates the inclusion rates of alternatively spliced IIIb and IIIc exons [28]. Work by others has further showed that additional proteins can act as “chromatin-adaptors” [29]–[32]. The question remains to what extent different chromatin adaptor complexes regulate splicing and which nucleosomes they interact with. A possible explanation of why the correlation of H3K36me3 with SEIR is diminished at the exon locus may lie in the position, relative to the exon, where different chromatin adaptors assemble and interact with H3K36me3 to regulate splicing. Further complicating the situation is a recent report demonstrating opposite causality, where alternative splicing was shown to modulate the levels of H3K36me3 enrichment [33], [34]. We observed no obvious spatial patterns between histone enrichment and splicing for lincRNAs (**Fig. 2B**).

Overall, our observations suggest that histone mark enrichment is associated with transcription start site exon inclusion and splicing and has a strong spatial signature. In addition to these analyses, we performed several control studies to establish that our results are genuine. First, our findings were robust even after controlling for gene expression, exon number, and genomic features such as simple repeats, microsatellites, and evolutionary conservation. Although the overall correlation between both TSSIR and SEIR and various histone modifications was moderate transcriptome-wide, the rapid change of correlation over short distances from exons and consistent patterns across multiple cell lines suggest an authentic relationship.

### TSSIR, SEIR, and histone marks in cancer cells

Since cells accumulate many genetic and epigenetic aberrations during tumorigenesis [23], [35]–[37], normal and cancer cells may differ substantially in their epigenetic regulation of transcript diversity. To investigate this possibility, we studied whether the association between TSSIR, SEIR and histone modifications in cancer cell lines followed similar patterns as those observed in the normal cells. We thus repeated the analyses described above using the cancer cell line data and tested for significant differences between the results using normal and cancer cell data for both protein-coding genes as well as lincRNAs. Remarkably, protein-coding genes in cancer cell lines displayed very similar patterns of association between the TSSIR and histone modifications as normal cell lines; the histone marks H3K4me1, H3K4me2, H3K4me3, H3K9ac, H3K27ac, H3K79me2, and H2az, which we previously found to be highly correlated in normal cell lines, were also highly correlated with TSSIR in cancer cells (**Fig. 2C and 2D**). Their correlation profiles across upstream and downstream exon regions also did not significantly differ from those of normal cell lines (T-test, 0.13>*p*>0.89 across all −5 kb, −2 kb, −1 kb, 0 kb, 1 kb, 2 kb, and 5 kb regions). Similarly, the other comparisons between normal and cancer cells, for both protein-coding genes and lincRNAs, did not show significant differences either (see **Fig. 2** **,** **Table S1–S4 in Text S1, and Figure S5 in Text S1**). These findings imply that the same histone modifications are associated with transcript diversity in both normal and cancer cells and that perturbation of the epigenetic environment via experimental manipulation in normal cells would potentially be informative of cancer cells.

### Gene-specific association of histone mark enrichment with TSSIR and SEIR

**Figure 3:**
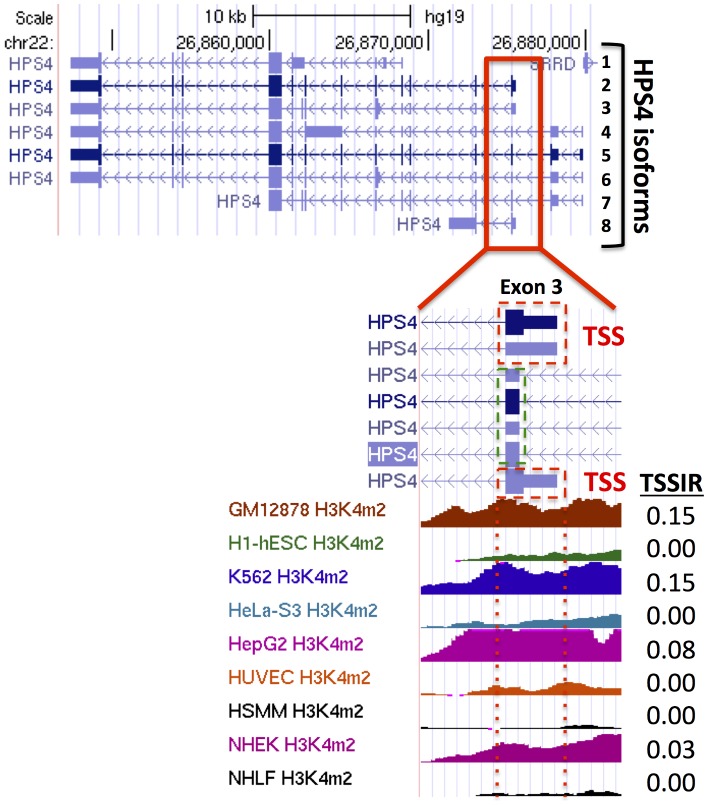
Differential H3K4me2 enrichment near exon 3 of HPS4. The HPS4 gene produces up to 8 isoforms. Three of these isoforms (isoforms 2, 3, and 8 – TSS exon marked with red dashed line) utilize the 3^rd^ exon as the transcription start site (TSS) and four isoforms (isoforms 4–7, exons marked with green dashed line) utilize the 3^rd^ exon as an internal exon. Considering the H3K4me2 modification within the exon 3 TSS boundaries (red dashed lines), the enrichment significantly differs between cell lines in which no isoforms with TSS at the 3^rd^ exon position (isoforms 2, 3, and 8) are expressed (EIR = 0) and cell lines that express isoforms with TSS at the 3^rd^ exon position.

So far, our transcriptome-wide and within-cell line approach identified an association between TSSIR, SEIR and histone enrichment across all exons but was unable to identify individual candidate genes with epigenetically regulated transcript diversity. We thus aimed to complement our investigation with a method that analyzes each exon individually across multiple cell lines. This approach is able to determine candidate genes with potential epigenetic regulation of transcript diversity and is analogous to an experimental setup where each cell line represents an experimental condition (i.e. varying levels of histone modification enrichment) resulting in a particular exon inclusion outcome. For example, the gene HPS4 (Hermansky-Pudlak syndrome gene 4) is expressed in all nine cell lines; its 3^rd^ exon is always excluded (SEIR = 0) in all HPS4 isoforms in H1hesc, Helas3, Hsmm, Huvec, and Nhlf cells, but is only occasionally included (0.03<SEIR<0.15) in Gm12878, Hepg2, K562, and Nhek cells (**Fig. 3**). Interestingly, the cell lines that always exclude this exon do not show a significant enrichment for H3K4me2 within exon boundaries (**Fig. 3**), whereas the remaining cell lines do and the difference between these two groups is significant (T-test, FDR-corrected p<0.003, **Methods**).

**Figure 4:**
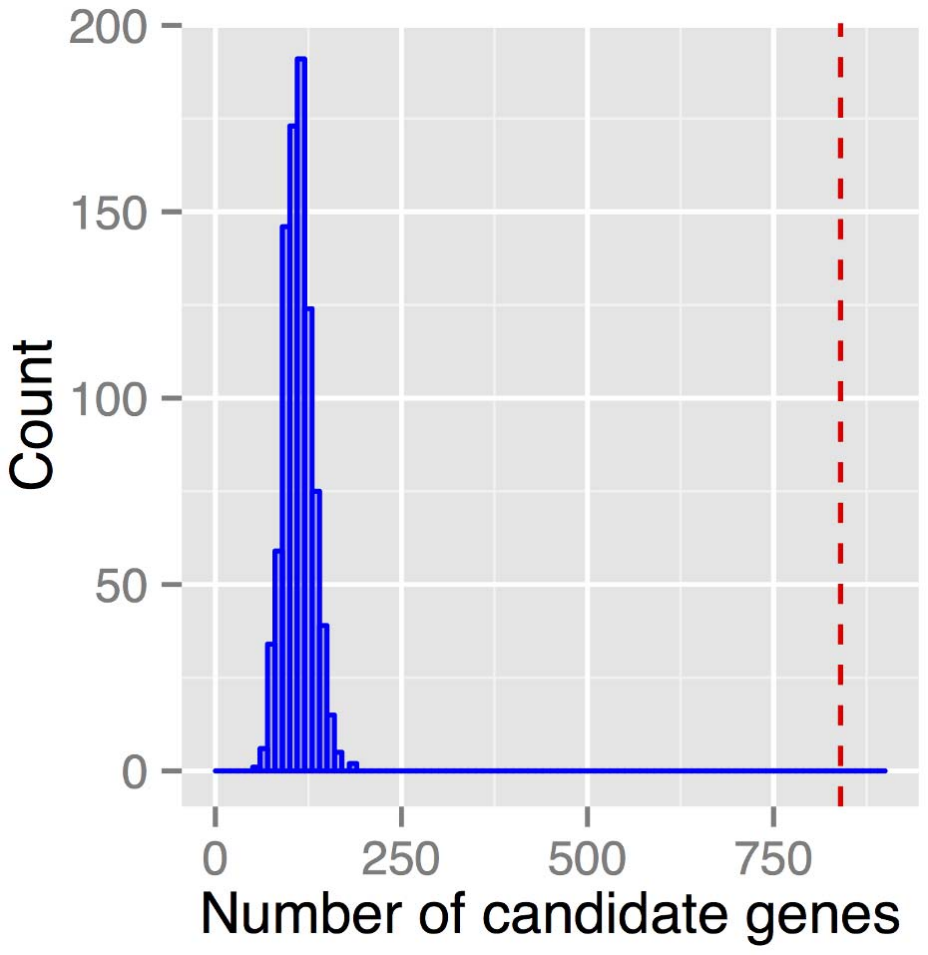
Number of significant genes expected by chance. We randomly reassigned exon TSSIR and SEIR labels, but left the same epigenetic background constant, and performed gene-specific analysis 1000 times to obtain a distribution of the number of significantly associated genes with transcript diversity (in blue). Observing 840 candidate genes in total (red horizontal line) was significantly higher (p<0.001) then expected by chance.

We thus analyzed all exons across all cell lines in a similar fashion, first only taking into account histone enrichment at the exon locus. Given the TSSIR and SEIR values across cell lines, each exon may be constitutively excluded (TSSIR = 0 and SEIR = 0), occasionally excluded (TSSIR>0 and SEIR<1), or retained (TSSIR = 1 and SEIR = 1). We then directly compared the histone modification levels for the inclusion pattern of a given exon across all available cell lines. The three possible two-way comparisons are: i) cell lines in which a given exon is always excluded versus retained (TSSIR = 0 vs. TSSIR = 1 or SEIR = 0 vs. SEIR = 1), ii) cell lines in which a given exon is retained versus occasionally excluded (TSSIR = 0 vs. 0<TSSIR<1 or SEIR = 0 vs. 0<SEIR<1), and iii) cell lines in which a given exon is occasionally excluded versus retained (0<TSSIR<1 vs. TSSIR = 1 or 0<SEIR<1 vs. SEIR = 1). Unfortunately, since the number of cell lines with available histone modification was limited, the power of this test was low. Nonetheless, given our stringent criteria (**Methods**), we identified 840 genes for which transcript diversity was significantly associated with histone modification enrichment at the exon locus (**Supplementary Dataset S1**). Specifically, 399 and 473 genes displayed a significant association between splicing and transcription start-site switching, respectively. Note that a single gene can be significant for the association between epigenetic patterning and both splicing and transcription start-site switching. To understand whether obtaining 840 candidate genes was a result of chance, we performed 1000 permutations by randomly reassigning exon labels for TSSIR and SEIR while keeping the epigenetic background of a gene constant. Observing 840 candidate genes in total was significantly higher (p<0.001) as compared to what was expected by chance (**Fig. 4**). These 840 genes were enriched for several GO terms (**Table S6 in Text S1**) including the regulation of the response to stimulus and development process. Thirty three of these genes were cancer-associated genes (**Supplementary Dataset S1**) (http://www.sanger.ac.uk/genetics/CGP/Census/).

### Histone modification enrichment predicts TSSIR and SEIR

We then aimed to predict exon inclusion patterns in an independent sample set. Specifically, given the histone enrichment levels and the inclusion pattern in the nine previously studied cell lines, we sought to determine, in independent cell lines, whether a given exon was always retained (SEIR = 1), always excluded (SEIR = 0), or occasionally excluded (0<SEIR<1) with regard to splicing or transcription start-site switching (TSSIR = 1, TSSIR = 0, or 0<TSSIR<1, respectively). These predictions were performed in the Hmec and Monocytes CD14 cell lines, for which more complete epigenetic information became available (http://genome.ucsc.edu/ENCODE/downloads.html). We limited our predictions to the 840 candidate genes identified above, since the cell lines previously analyzed provided evidence for an involvement of epigenetic marks in transcript diversity for only 840 candidate genes; attempting to predict exon inclusion based on epigenetic information for genes that are not epigenetically regulated would thus not be appropriate.

**Figure 5:**
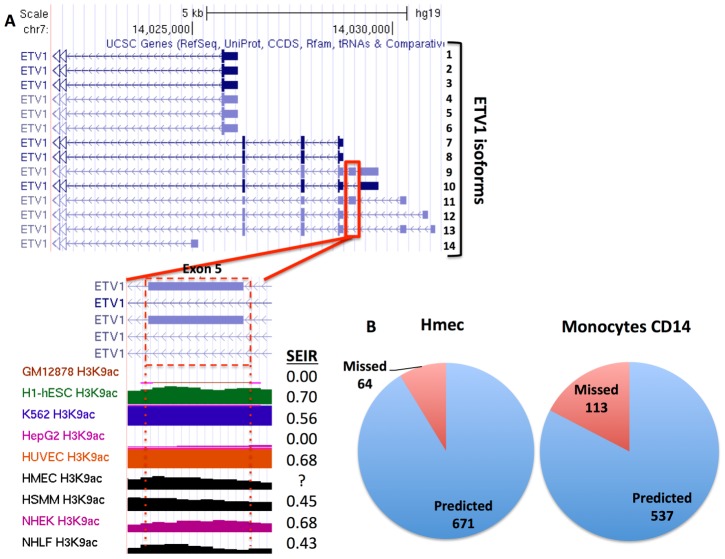
Prediction of ETV1 exon 5 inclusion in the Hmec cell line and overall prediction accuracy. (**A**) Exon 5 of ETV1 is present in isoforms 9 and 11, but it is spliced in isoforms 10, 12, and 13. Comparing the enrichment of H3K9ac, cell lines from which exon 5 was constitutively spliced (Gm12878 and HepG2) displayed an absence of H3K9ac, whereas the remaining cell lines, including Hmec, showed varying levels of H3K9ac enrichment. Since the Hmec cell line H3K9ac enrichment resembles that of the cell lines in which the 5^th^ exon was not constitutively spliced out, we predicted that exon 5 in Hmec would only occasionally be excluded. (**B**) The numbers of exons for which the inclusion pattern was correctly vs. incorrectly predicted in Hmec and Monocytes CD14 cell lines.

**Figure 6:**
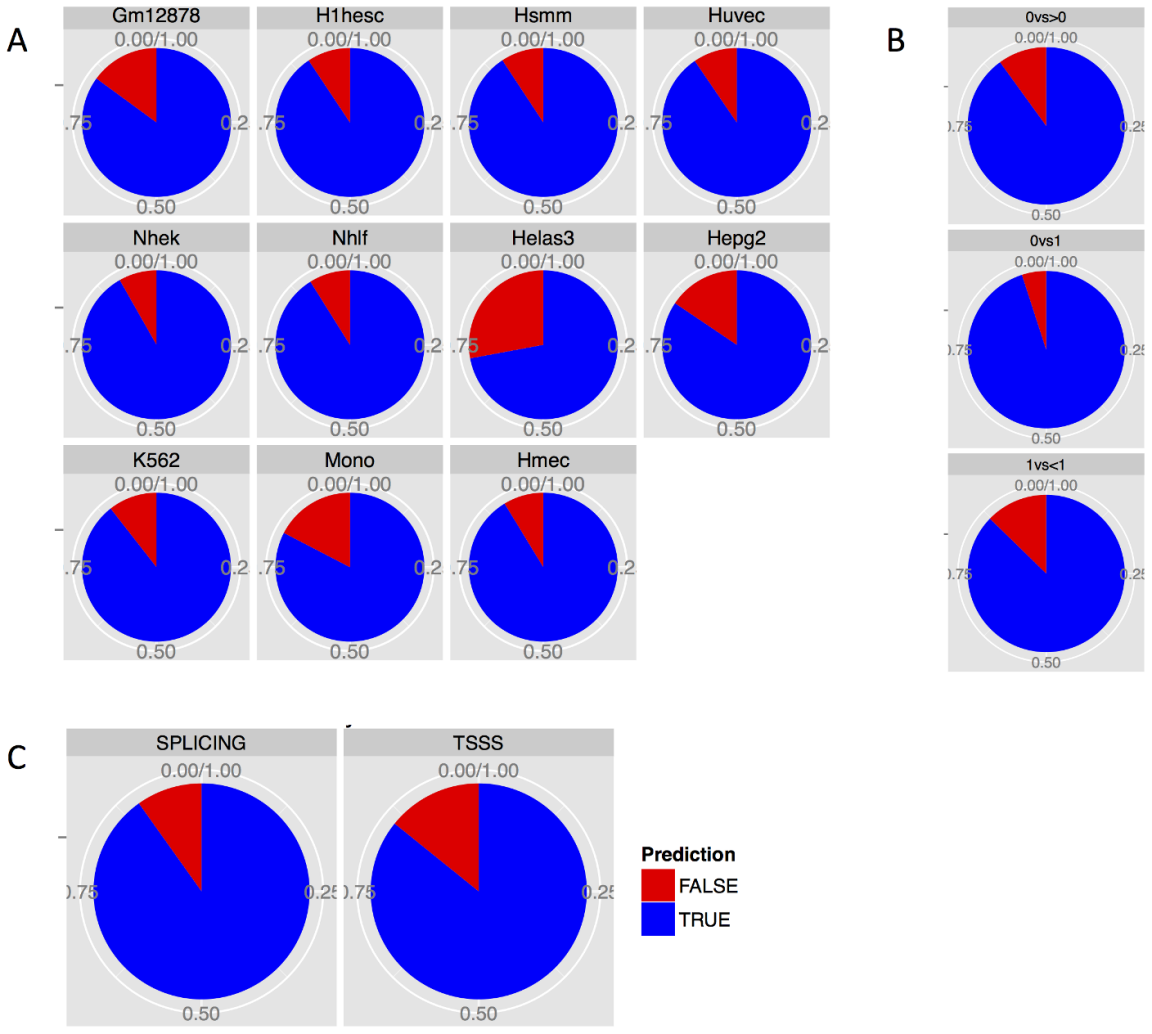
Prediction accuracy. (**A**) Prediction accuracy of exon inclusion categories from leave-one-out cross-validation by cell line being predicted. (**B**) Prediction accuracy by exon inclusion category comparison. (**C**) Prediction accuracy of exon inclusion categories for splicing (“SPLICING”) and transcription start-site switching (“TSSS”).

To illustrate our approach, consider exon 5 of the ETV1 gene in the Hmec cell line; for this exon, we generated a matrix containing enrichment values for all histone modifications, which were significantly associated with SEIR (in this case H3K9ac, H3K4me3, H3K4me2, and H3K27ac) for the original cell line set (Gm12878, Hsmm, Huvec, Hepg2, Helas3, K562, H1hesc, Nhek, and Nhlf), and identified the SEIR of this exon in each cell line. All ETV1 isoforms in Gm12878 and Hepg2 cell lines lacked exon 5 (SEIR = 0) whereas some isoforms expressed in H1hesc, Hsmm, Huvec, K562, Nhek, and Nhlf cell lines contained exon 5 (SEIR range 0.43–0.69) (**Fig. 5A**). The difference in histone enrichment between these groups was striking: the Gm12878 and Hepg2 cell lines completely lacked enrichment in H3K9ac, H3K4me3, H3K4me2, and H3K27ac while the remaining cell lines were strongly enriched in those marks (**Fig. 5A**). We then calculated the pairwise Euclidean distance between all cell lines and the first validation line, Hmec, and determined the three nearest-neighbor cell lines signified by the smallest Euclidean distance (**Methods**). Since Hmec was enriched for all four histone marks in exon 5, its epigenetic profile was closest to that of the Nhlf, K562, and Huvec cell lines. We therefore predicted that in Hmec, exon 5 of ETV1 was occasionally excluded from some fraction of isoforms (0<SEIR<1), which was validated by the finding that in this cell line, SEIR = 0.74. When extending this approach to all candidate genes, we predicted the correct exon inclusion category with an accuracy of 91.82% and 84.65% for Hmec and Monocytes CD14 cell lines, respectively (**Fig. 5B**). To establish whether such high prediction accuracy can be established across all cell lines, we performed leave-one-out cross-validation following the approach described above. The accuracies for individual cell lines ranged from 72.1% in the Helas3 cell line to 91.8% in the Nhek cell line, with an average accuracy of 87.2% (**Fig. 6**). We also calculated the overall accuracy separately for splicing and for transcription start-site switching, which was 90.16% and 85.81%, respectively. Although the 0<EIR<1 vs. EIR = 1 comparison is the most frequent (76%), the accuracy for all comparisons consistently were high, at 90.00%, 95.00%, and 87.28% for EIR = 0 vs. 0<EIR<1, EIR = 0 vs. EIR = 1, and 0<EIR<1 vs. EIR = 1, respectively. Details regarding the fraction of genes that could be assigned into comparative groups and the number of significant genes for each validation step are displayed in **Table S7 in Text S1**. These findings suggest that the histone modification enrichment levels alone can be used to predict the inclusion pattern of an exon.

## Discussion

In this study, we analyzed the association between transcription start-site switching, spliced exon inclusion rates and histone modification patterns across multiple normal and cancer cell lines for protein-coding genes and lincRNAs. Unlike previous studies [8], [16], [17], which established the relationship between epigenetic patterning and gene expression levels, we addressed the association of the epigenetic background of a gene with its transcript isoform diversity. The main difference between ours and previous investigations therefore is that our study investigates relative isoform diversity of expressed genes, and not actual expression levels.

We used two approaches to address this issue. The first approach correlated transcriptome-wide (ie “within cell line”) transcription start site inclusion rates and spliced exon inclusion rates with histone enrichment levels. The second approach investigated gene-specific associations between transcription start site inclusion rates, spliced exon inclusion rates and histone enrichment levels. The shortcomings and assumptions made by each method are discussed below. Overall, our study led to four main findings. (i) The role of epigenetic patterning in transcription start-site switching is likely to be common across the genome for both protein-coding genes as well as lincRNAs. (ii) The role of epigenetic patterns in splicing is likely gene-specific, with the exception of H3K36me3 (discussed below). (iii) Our gene-specific approach led to the identification of 840 candidate genes whose exon inclusion rates for transcription start-site switching and splicing were strongly associated with patterns of histone modifications. (iv) Lastly, histone modification data alone can be used to predict the inclusion pattern of an exon.

Our first and second findings are based on the observation that both transcriptome-wide and gene-specific approaches identified a common set of histone marks that were associated with transcription start-site switching (H3K4me1, H3K4me2, H3K4me3, H3K9ac, H3K27ac, and H2az), whereas the results of these two methods differed for the case of splicing. Transcriptome-wide analysis for splicing showed a pronounced association of splicing inclusion rates with H3K36me3 whereas the gene-specific approach identified H2az, H3K4me1, H3K4me2, H3K4me3, H3K9ac, H3K9me3, H3K27ac, H3K27me3, H3K36me3, H3K79me2, and H4K20me1 as significantly associated marks. This discrepancy is likely a result of a bias by the transcriptome-wide approach to detect common genome-wide trends and the gene-specific approach to identify unique relationships for each exon.

Observing both common and gene-specific histone marks associated with splicing is in line with the proposed models of epigenetic regulation of splicing: the kinetic model and the chromatin-adaptor model [38]. According to the kinetic model, chromatin structure affects the elongation rate of RNA polymerase, which in turn influences the competition between weak and strong splice sites for the recruitment of splicing factors [38]. The chromatin-adaptor model, on the other hand, describes an interaction between specific histone marks and pre-mRNA molecules through a chromatin-adaptor complex, which aids in the recruitment of splicing factors to pre-mRNA splicing sites [26], [30], [39]. Since these two models are not mutually exclusive, one can imagine H3K36me3, known to be associated with transcription elongation [16], [17], to act as a common factor in splicing genome-wide, while other histone marks can act in a gene-specific manner. Interestingly, histone marks traditionally associated with transcription initiation and transcription repression, such as H3K4me3 and H3K9me3, respectively, were also found in our study to be associated with splicing gene-specifically. This observation is in line with experimental studies describing splicing chromatin-adaptor complex for H3K4me3 [40] and for H3K9me3 [41]. Further extending the realm of epigenetic regulation of transcript diversity is a recent work by Mercer and colleagues, which presented evidence for the role of 3-dimensional DNA conformation in splicing [42]. According to this study, exons sensitive to DNase I are spatially located close to transcription factories near promoter regions containing initiating Pol II as well as other general transcription and splicing factors.

**Figure 7:**
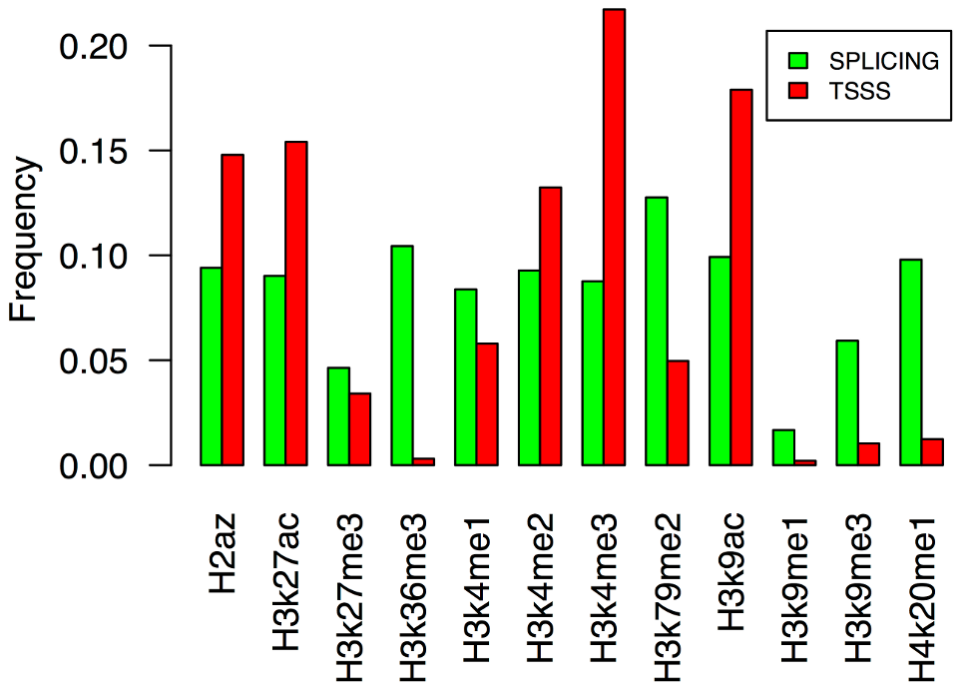
Frequency of histone modification marks found significantly associated with transcription start-site switching (marked red) and splicing (marked green). Our gene-specific approach identified 840 candidate genes for which transcript diversity significantly associated with histone modification enrichment. Note that transcription start-site switching and splicing of a single gene can be associated with multiple histone marks.

Interestingly, a large fraction of alternatively spliced exons are DNase I sensitive [42]. This finding suggests that the epigenetic background of an exon cannot only interact with splicing factors via chromatin adaptor complexes, but potentially also induce 3-dimensional DNA conformation changes that enhance the likelihood of interactions with general transcription factors, and perhaps thus influence the splicing frequency. This 3-dimensional conformation is likely enhanced via particular sets of histone modifications. Interestingly, our second analysis, testing individual exon across all cell lines, revealed that alternatively spliced exons were frequently associated with different enrichment levels of histone marks well known to be associated with promoters and enhancers, such as H3K4me1, H3K4me2, H3K4me3, H3K27ac, and H3K9ac [16], [17] (**Fig. 7**). Accounting for such a 3-dimensional model could further strengthen the association found between histone modification enrichment and transcription start-site switching and splicing,

There are however shortcomings to both approaches. The transcriptome-wide method makes two assumptions that may be violated in cells. First, correlating transcription start site inclusion rates and spliced exon inclusion rates with histone mark enrichment assumes that (i) transcript diversity of all genes is associated with their epigenetic background, and additionally (ii) these rates are associated with the same histone modification. Likely, it is for these reasons that the correlations between exon inclusion rates and histone mark enrichment are rather moderate. As mentioned above, however, because of the rapid change of these correlations over short distances from exons and the consistent patterns across multiple cell lines, these associations suggest a genuine relationship. The shortcoming of the gene-specific approach lies in the low statistical power of eleven cell lines analyzed and the natural tendency to miss tissue specific exon behavior. This is particularly the case for lincRNAs, of which 30%, according to recent estimates, have tissue specific expression [2].

Since a large body of experimental data indicates that aberrant splicing of gene transcripts significantly contributes to many areas of cancer biology, including metabolism, apoptosis, cell cycle control, invasion and metastasis [43]–[45], it is imperative to further our understanding of the regulatory and/or potentially disruptive role of epigenetic patterning in alternative splicing and transcription start site selection in tumorigenesis. Significant effort has been devoted to the discovery of DNA aberrations that drive cancer progression [35]–[37], [46]; surprisingly, however, there is only a small number of recurrent genomic changes within and across cancer types, with few prominent exceptions [47]–[49]. While the identification of affected pathways rather than individual genes affected by DNA mutations might lead to more informative results, the possibility remains that aberrant phenotypes in cancer are largely driven by the epigenetic component of gene expression and transcript deregulation [23], [50], [51]. Our study identified several histone modifications (H3K4me1, H3K4me2, H3K4me3, H3K9ac, H4K27ac, H3K36me3, and H3K79me2) that are strongly associated with transcript diversity across multiple independent cell types as well as 840 candidate genes for which there is evidence of epigenetic co-regulation of transcript diversity. Our work represents a step towards identifying the functional consequences of histone modifications on transcript diversity and suggests a rational methodology for the analysis of modern, large-scale datasets, which can be applied to any sample sets.

## Methods

### Data sets analyzed

#### Cell lines

We analyzed RNA-seq data from nine human cell lines (Gm12878, Hsmm, Huvec, Hepg2, Helas3, K562, H1hesc, Nhek, Nhlf) (http://genome.ucsc.edu/ENCODE/), of which six were normal (Gm12878, Hsmm, Huvec, H1hesc, Nhek, Nhlf) and three were cancer cell lines (Hepg2, Helas3, K562). For all nine cell lines, we obtained information of the genome-wide patterns of the following twelve histone marks: H3K4me1, H3K4me2, H3K4me3, H3K9ac, H3K9me1, H3K9me3, H4K20me1, H3K27ac, H3K27me3, H3K36me3, H3K79me2, H2az (**Table 1**).

#### RNA-seq data

RNA-seq data (paired-end 75 nt reads) from nine human cell lines (Gm12878, Hsmm, Huvec, Hepg2, Helas3, K562, H1hesc, Nhek, Nhlf) was downloaded from the UCSC ENCODE database (http://genome.ucsc.edu/ENCODE/downloads.html) and used to calculate relative exon expression. Prior to mapping reads to the human genome (hg19), each fastq file was processed in the following way: quality score statistics at all nucleotide positions for all fastq files were obtained using a python script [52] from GALAXY (https://main.g2.bx.psu.edu/). All reads in each fastq file were trimmed at the same position, at which the second lowest quartile quality score dropped below 20. This procedure resulted in read lengths of about 50–70 bp, depending on the quality of the sequencing run. Multiple replicates were pooled and analyzed together. Read alignment was performed using the TopHat software package [24], which is an alignment tool optimized for mapping reads across exon-exon junctions. During the alignment step, we allowed for one mismatch between the read and the genome and used UCSC gene annotation (hg19) as a guiding gene model set to accommodate for lower quality bias toward the 3′ read end but also to maintain a nearly identical match to the genome. The TopHat output was then further processed with Cufflinks software [25], which assembles transcript isoforms and quantifies isoform expression. Cufflinks was run with the option of assembling only those transcript isoforms that are strictly supported by a given gene annotation (UCSC hg19).

#### ChIP-seq data

In order to assess the level of histone modifications across all exons, we analyzed pre-computed bam files from ENCODE ChIP-seq experiments [17], [53] for 12 histone marks (H3K4me1, H3K4me2, H3K4me3, H3K9ac, H3K9me1, H3K9me3, H4K20me1, H3K27ac, H3K27me3, H3K36me3, H3K79me2, H2az). Since the genomic regions of interest had known boundaries (*i.e.* exon coordinates), we directly counted the number of overlapping reads with a given genomic region to attain the raw signal. The minimum overlap between a read and a genomic location was set to one nucleotide.

### Determination of the exon inclusion rate

Exons were grouped into four categories: transcription start site, internal, transcription end site, or overlapping exons. We then quantified the presence of each exon type. Only internal and transcription start site exons were used for further analysis. The relative presence of transcription start site exons (TSSIR – transcription start site inclusion rate) and spliced exons (SEIR – splicing exon inclusion rate) was calculated from the Cufflinks .gtf output file and reflects the fraction of all isoforms from a given gene that contain a given exon. The inclusion rates therefore have ranges of 0<TSSIR≤1 and 0≤SEIR≤1.

### Determination of histone modification enrichment

Using raw signal read counts of histone marks and reference samples (input DNA) for each cell line, we calculated the presence of histone mark enrichment using a Fisher's test statistic and considered enrichment significant [17] if *p*<0.0001. The level of enrichment was calculated as 
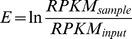
. *RPKM* is defined as 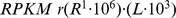, where *r* represents the number of reads mapped to a given exon, *R* is the total number of reads mapped, and *L* defines the length of a given exon. *RPKM* therefore denotes the number of reads per kilobase of exon per million reads mapped.

### Association between histone modifications and SEIR as well as TSSIR

Prior to further analysis, we filtered our exon set to contain only internal exons and exons of genes that express more than one isoform in at least one normal or cancer cell line. In order to avoid potential problems with mapping RNAseq reads to closely related genes, we further excluded genes with paralogs more than 95% identical on the DNA level to generate the final curated exon dataset. We then calculated the Spearman rank correlation (which is more robust for asymmetrical distributions of TSSIR and SEIR and a large fraction of ties than Pearson's correlation) between TSSIR and SEIR and histone enrichment values, excluding all exons for which Fisher's test for histone enrichment was not significant (p>0.0001).

### Spatial patterns of correlations

To assess the spatial patterns of correlation between histone modifications and SEIR as well as TSSIR, we calculated the extent of histone enrichment inside 1 kb, 2 kb, and 5 kb blocks immediately upstream or downstream of exons. The upstream 1 kb, 2 kb, and 5 kb regions extended from the upstream exon coordinate a given distance whereas the downstream 1 kb, 2 kb, and 5 kb regions extended from the downstream exon coordinate for a given distance. The Spearman rank correlation was then determined between each upstream or downstream block and the corresponding exon TSSIR or SEIR.

### Fisher transformation of correlation coefficients

To allow for direct comparisons between correlation coefficients of different cell lines and histone modifications, we transformed the Spearman *p* using the Fisher transformation formula, 
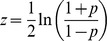
.

### Identification of candidate genes with epigenetically regulated transcript diversity

To identify genes with epigenetically regulated transcript diversity, we analyzed each exon in the context of the nine cell lines (Gm12878, Hsmm, Huvec, Hepg2, Helas3, K562, H1hesc, Nhek, Nhlf). We categorized the exon inclusion rate into three groups: SEIR = 0, 0<SEIR<1, and SEIR = 1. We followed the same approach for TSSIR. Next, we tested whether any histone modification displayed a statistically significant difference in its enrichment in any possible two-group comparison, given an exon's SEIR values across the nine cell lines. For example, if the pattern of SEIR values for a given exon allowed us to separate the nine cell lines into two groups that showed either SEIR = 0 or SEIR = 1, we used T-test to determine whether the respective histone modification enrichment among the two groups of cell lines was statistically different. All *p*-values were corrected for false discovery rate (FDR) [54]. To discover cell-specific events, we allowed for comparisons where only one cell line versus many could be assigned to an SEIR or TSSIR group. Naturally, given the lower power of this test, most of these did not pass our 5% FDR cutoff. This approach identified 840 genes, for which at least one exon showed a statistically significant association between SEIR and at least one histone modification (ie. statistically significant difference in histone modification enrichment between two SEIR groups for a given exon).

### Prediction of exon exclusion or retention with histone modification enrichment

We limited our predictions of exon exclusion or retention in the Hmec and Monocytes CD14 cell lines to the 840 candidate genes that showed significant association between the TSSIR or SEIR and histone modification enrichment in the original set of nine human cell lines (Gm12878, Hsmm, Huvec, Hepg2, Helas3, K562, H1hesc, Nhek, Nhlf). For a given exon, we constructed a Euclidean distance matrix with the formerly identified set of histone modifications for all cell lines, including Hmec and Monocytes CD14. Next, we determined the three closest neighbors of Hmec and Monocytes CD14 from among the original set of nine cell lines (Gm12878, Hsmm, Huvec, Hepg2, Helas3, K562, H1hesc, Nhek, and Nhlf). Because the exon inclusion rates for a given exon were known in the original set of nine cell lines, we separated these cell lines into three comparison groups: i) cell lines in which a given exon was always excluded versus retained (SEIR = 0 vs. SEIR = 1), ii) cell lines in which a given exon was retained versus occasionally excluded (SEIR = 0 vs. 0<SEIR<1), and iii) cell lines in which a given exon was occasionally excluded versus retained (0<SEIR<1 vs. SEIR = 1). The inclusion status - retained, occasionally excluded, or always excluded - of a given exon in the Hmec or Monocytes CD14 cell lines was then determined based on what comparison group the majority of the three closest neighbors belonged to. For example, if the majority of Hmec's three closest neighbors (based on the Euclidian distance matrix) belonged to the group SEIR = 1, then we would predict that particular exon in the Hmec cell line was always retained, ie. a given gene was expressing only those isoforms that included our exon of interest. We applied the same approach to transcription start site exons and their respective TSSIR values.

### Identification of epigenetically conserved and divergent regions in cancer

#### Epigenetic conservation

Cancer cells often undergo dramatic epigenetic reprogramming [23], [51]; we therefore aimed to identify genes residing in epigenetically aberrant regions as well as the positional effect of a gene on its splicing patterns. We divided the genome into 100 kb non-overlapping blocks; we excluded all 100 kb blocks spanning across UCSC coordinates of centromeric or telomeric regions, and obtained a total of 32,433 blocks. We then determined the extent of epigenetic conservation of each block and each histone modification in the following way: since conserved regions are expected to have very similar histone enrichment levels across multiple cell lines, for each 100 kb block, we calculated an index of dispersion, 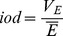, where *V_E_* represents the histone enrichment variance across normal cell lines, and *E* is the enrichment mean of normal cell lines (Gm12878, Hsmm, Huvec, H1hesc, Nhek, Nhlf). Regions lacking any enrichment were excluded from the subsequent quantile analysis. We identified those 100 kb blocks with the lowest quartile index of dispersion (*iod*) as epigenetically conserved regions. Next, we compared these epigenetically conserved regions in normal cell lines to each of the cancer cell lines (Helas3, Hepg2, K562) and calculated a Z statistic. In a given cancer cell line, genomic regions with the lowest quartile absolute value Z score were deemed “conserved” whereas regions with the highest quartile absolute value Z score were identified as “divergent”. This approach identified between 11,285 and 22,506 conserved and between 616 and 1410 divergent regions given a particular histone modification.

#### Gene enrichment analysis

To assess whether epigenetically perturbed regions in cancer cell lines harbored cancer-associated genes, we performed a gene enrichment analysis. We utilized the cancer gene census (COSMIC) maintained by the Welcome Trust Sanger Institute (http://www.sanger.ac.uk/genetics/CGP/Census/) for a curated list of known cancer associated genes. For each cancer cell line and histone modification, we then performed a hypergeometric test on the enrichment of cancer-associated genes in epigenetically divergent regions. Taking into consideration all cancer-related genes classified in the COSMIC database, we found that regions with a significant enrichment of H3K4me3 and H3K79me2 displayed an enrichment for cancer-associated genes. For example, regions that were aberrant in H3K4me3 in cancer cells as compared to normal cells were enriched for cancer-associated genes in the Helas3 and K562 cell lines (hypergeometric test, *p* = 0.023 and *p* = 0.009 respectively, **Table S8 in Text S1**). Similarly, aberrant regions for H3K79me2 were enriched for cancer-associated genes in the Hepg2 and K562 cell lines (*p* = 0.040 and *p*<0.001, respectively).

## Supporting Information

Dataset S1 This workbook contains 11 sheets with leave-one-out cross-validation candidate genes and 1 sheet with a union of all 840 candidate genes from leave-one-out cross-validations. Individual cross-validation candidate gene data is named after the cell line analyzed. P-values are corrected for multiple testing using FDR. Comparison categories 0 vs 1, 0 vs >0, and 1 vs <1 represent combinations tested given the scenario, where each exon may be constitutively excluded (TSSIR = 0 and SEIR = 0), occasionally excluded (TSSIR>0 and SEIR<1), or retained (TSSIR = 1 and SEIR = 1). The “SplicingOrTsss” column then differentiates whether a given comparison category corresponds to transcription start site switching or splicing. Note that column “Statistic” frequently contains value “-Inf”; this refers to the case for comparisons where one of the groups has an enrichment of 0, ie. no histone mark enrichment.
(XLSX)

Text S1 Supporting Materials. Includes: Module S1 in Text S1. Epigenetically aberrant regions in three cancer cell lines are enriched for oncogenes; Table S1 in Text S1. Association between the transcription start site inclusion rate (TSSIR) of lincRNAs and histone modification enrichment in normal cell lines; Table S2 in Text S1. Association between splicing exon inclusion rate (SEIR) of protein coding genes and histone modifications in cancer cell lines; Table S3 in Text S1. Association between transcription start site inclusion rate (TSSIR) of lincRNAs and histone modification enrichment in cancer cell lines; Table S4 in Text S1. Association between splicing exon inclusion rate (SEIR) of lincRNAs and histone modifications in cancer cell lines; Table S5 in Text S1. Association between histone modification enrichment and transcription start site inclusion rate; Table S6 in Text S1. Top 20 ontology categories enriched among 840 candidate genes that showed a significant association between splicing exon inclusion rates and histone modification enrichment; Table S7 in Text S1. Leave-one-out cross validation summary statistics.
(DOC)
