# Supplementary material for: Histone Modifications Are Associated with Transcript Isoform Diversity in Normal and Cancer Cells
Source: PLoS Comput Biol. 2014 Jun 5;10(6):e1003611. doi: 10.1371/journal.pcbi.1003611 (PMC4046914; doi:10.1371/journal.pcbi.1003611)
Supplement: Text S1 — Supporting Materials. Includes: Module S1 in Text S1. Epigenetically aberrant regions in three cancer cell lines are enriched for oncogenes; Table S1 in Text S1. Association between the transcription start site inclusion rate (TSSIR) of lincRNAs and histone modification enrichment in normal cell lines; Table S2 in Text S1. Association between splicing exon inclusion rate (SEIR) of protein coding genes and histone modifications in cancer cell lines; Table S3 in Text S1. Association between transcription start site inclusion rate (TSSIR) of lincRNAs and histone modification enrichment in cancer cell lines; Table S4 in Text S1. Association between splicing exon inclusion rate (SEIR) of lincRNAs and histone modifications in cancer cell lines; Table S5 in Text S1. Association between histone modification enrichment and transcription start site inclusion rate; Table S6 in Text S1. Top 20 ontology categories enriched among 840 candidate genes that showed a significant association between splicing exon inclusion rates and histone modification enrichment; Table S7 in Text S1. Leave-one-out cross validation summary statistics. (DOC) [file pcbi.1003611.s002.doc]

**Supplementary Information**

**Histone modifications are associated
with transcript isoform diversity in normal and cancer cells**

Ondrej Podlaha^1^, Subhajyoti De^2,3,4^, Mithat Gonen^5^, and Franziska Michor^1*^

^1^Department of Biostatistics and Computational Biology, Dana-Farber Cancer Institute,
and Department of Biostatistics, Harvard School of Public Health, Boston, MA 02215, USA.

^2^Department of Medicine, University of Colorado School of Medicine, Aurora, CO 80045, USA. ^3^Department of Biostatistics and Informatics, Colorado School of Public Health, Aurora, CO 80045, USA. ^4^Molecular Oncology Program, University of Colorado Cancer Center, Aurora, CO 80045, USA. ^5^Department of Epidemiology and Biostatistics, Memorial Sloan-Kettering Cancer Center, New York, NY 10065, USA. ^*^Author for correspondence. Department of Biostatistics and Computational Biology, Dana-Farber Cancer Institute, 450 Brookline Avenue, Boston, MA 02215, USA. Tel: 617 643 5045. Fax: 617 632 2444. Email: [michor@jimmy.harvard.edu](mailto:michor@jimmy.harvard.edu).

^*^Author for correspondence.

**Table S1. Association between the transcription start site inclusion rate (TSSIR) of lincRNAs and histone modification enrichment in normal cell lines.** We analyzed six normal human cell lines (Gm12878, Hsmm, Huvec, H1hesc, Nhek, Nhlf) for the associations between the transcription start site inclusion rate (TSSIR) and histone modification enrichment for lincRNAs. Values represent the average of Fisher transformed Spearman rank correlations to enable direct comparison. Coefficients are color-coded, with red representing increasingly negative and green representing increasingly positive correlation. Distance from exon categories signifies a region relative to a given exon where histone enrichment was measured. 0kb represents a region within given exon boundaries, and 1kb, 2kb, and 5kb signify regions from the exon boundary either upstream (negative) or downstream (positive).

**Table S2. Association between splicing exon inclusion rate (SEIR) of protein coding genes and histone modifications in cancer cell lines.** We analyzed three cancer cell lines (Hepg2, Helas3, K562) for associations between splicing exon inclusion rate (SEIR) and histone modification enrichment for protein coding genes. Values represent the average of Fisher transformed Spearman rank correlations to enable direct comparison. Coefficients are color-coded, with red representing increasingly negative and green representing increasingly positive correlation. Distance from exon categories signifies a region relative to a given exon where histone enrichment was measured. 0kb represents region within given exon boundaries, and 1kb, 2kb, and 5kb signify regions from the exon boundary either upstream (negative) or downstream (positive).

**Table S3. Association between transcription start site inclusion rate (TSSIR) of lincRNAs and histone modification enrichment in cancer cell lines.** We analyzed three cancer cell lines (Hepg2, Helas3, K562) for associations between transcription start site inclusion rate (TSSIR) and histone modification enrichment for lincRNAs. Values represent the average of Fisher transformed Spearman rank correlations to enable direct comparison. Coefficients are color-coded, with red representing increasingly negative and green representing increasingly positive correlation. Distance from exon categories signify region relative to a given exon where histone enrichment was measured. 0kb represents region within given exon boundaries, and 1kb, 2kb, and 5kb signify regions from the exon boundary either upstream (negative) or downstream (positive).

**Table S4. Association between splicing exon inclusion rate (SEIR) of lincRNAs and histone modifications in cancer cell lines.** We analyzed three cancer cell lines (Hepg2, Helas3, K562) for associations between splicing exon inclusion rate (SEIR) and histone modification enrichment for protein coding genes. Values represent the average of Fisher transformed Spearman rank correlations to enable direct comparison. Coefficients are color-coded, with red representing increasingly negative and green representing increasingly positive correlation. Distance from exon categories signifies a region relative to a given exon where a histone enrichment was measured. 0kb represents region within given exon boundaries, and 1kb, 2kb, and 5kb signify regions from the exon boundary either upstream (negative) or downstream (positive).

**Table S5. Association between histone modification enrichment and transcription start site inclusion rate.** Correlations between transcription start site inclusion rate or splicing and enrichment of selected histone modifications in normal or cancer cell lines. The following correlations and cell line categories are: **(A)** transcription start site switching and normal cell lines, **(B)** splicing and normal cell lines, **(C)** transcription start site switching and cancer cell lines, and **(D)** splicing and cancer cell lines. Black dots represent median Spearman rank correlations between exon inclusion rate and given histone marks. All correlation coefficients were transformed using a Fisher’s transformation before plotting. Notches were calculated as $\pm1.58\times\left(\frac{IQR}{\sqrt{n}}\right)$ where *IQR* stands for inter quartile range and *n* for sample size. Distances from exon represent genomic blocks of a given size from exon start (upstream regions) or exon end (downstream regions).

**A**

**B**

**C**

**D**

**Table S6. Top 20 ontology categories enriched among 840 candidate genes that showed a significant association between splicing exon inclusion rates and histone modification enrichment.**

**Table S7. Leave-one-out cross validation summary statistics.**

**Module S1. Epigenetically aberrant regions in three cancer cell lines are enriched for oncogenes.** Using the cancer gene consensus from COSMIC, we tested for oncogene enrichment in epigenetically aberrant regions of three cancer cell lines (Helas3, Hepg2, and K562) with regard to specific histone marks using the hypergeometric test. All *p* values were corrected for multiple testing (FDR). Green highlights significantly enriched marks at the 5% FDR level.
